# Causes and Prognosis of Intestinal Failure in Crohn’s Disease: An 18-year Experience From a National Centre

**DOI:** 10.1093/ecco-jcc/jjaa060

**Published:** 2020-03-26

**Authors:** Mattias Soop, Haroon Khan, Emma Nixon, Antje Teubner, Arun Abraham, Gordon Carlson, Simon Lal

**Affiliations:** Irving National Intestinal Failure Unit, University of Manchester, Manchester Academic Health Science Centre, Salford Royal NHS Foundation Trust, Manchester, UK

**Keywords:** Intestinal failure, parenteral nutrition, surgery, optimisation

## Abstract

**Background and Aims:**

Intestinal failure [IF] is a feared complication of Crohn’s disease [CD]. Although cumulative loss of small bowel due to bowel resections is thought to be the dominant cause, the causes and outcomes have not been reported.

**Methods:**

Consecutive adult patients referred to a national intestinal failure unit over 2000–2018 with a diagnosis of CD, and subsequently treated with parenteral nutrition during at least 12 months, were included in this longitudinal cohort study. Data were extracted from a prospective institutional clinical database and patient records.

**Results:**

A total of 121 patients were included. Of these, 62 [51%] of patients developed IF as a consequence of abdominal sepsis complicating abdominal surgery; small bowel resection, primary disease activity, and proximal stoma were less common causes [31%, 12%, and 6%, respectively]. Further, 32 had perianastomotic sepsis, and 15 of those had documented risk factors for anastomotic dehiscence. On Kaplan-Meier analysis, 40% of all patients regained nutritional autonomy within 10 years and none did subsequently; 14% of patients developed intestinal failure-associated liver disease. On Kaplan-Meier analysis, projected mean age of death was 74 years.^2^

**Conclusions:**

IF is a severe complication of CD, with 60% of patients permanently dependent on parenteral nutrition. The most frequent event leading directly to IF was a septic complication following abdominal surgery, in many cases following intestinal anastomosis in the presence of significant risk factors for anastomotic dehiscence. A reduced need for abdominal surgery, an increased awareness of perioperative risk factors, and structured pre-operative optimisation may reduce the incidence of IF in CD.

## 1. Introduction

Intestinal failure [IF] is a feared complication of Crohn’s disease [CD].^1^ Intestinal failure is defined as a reduction of gut function below the minimum necessary for the absorption of macronutrients, water, or electrolytes, such that parenteral supplementation is required.^2^ Recent reports from national intestinal failure units in the UK show that CD is the underlying disease in 30–32% of patients with long-term intestinal failure.^3,4^ Longitudinal data from Japan indicate that the incidence of IF in CD is 8.5–18.2% during the first 20 years after initial presentation.^5,6^

The mechanisms of IF in CD are poorly understood. Short bowel syndrome due to repeated small bowel resections is the mechanism that is most prominently quoted in textbooks and current clinical guidelines.^7–9^ Bowel-sparing techniques such as strictureplasty and endoscopic balloon dilatation have been developed specifically in order to minimise loss of small bowel and reduce the risk of intestinal failure.^10,11^ However, although bowel-sparing techniques are undoubtedly important in intestinal surgery, there is no evidence that intestinal resection is the predominant mechanism of IF in CD. In contrast, in our previous smaller study we found that of 41 patients with IF secondary to CD, successive small bowel resection was the cause in only a minority [22%] of patients.^12^ In the majority [61%], IF developed as a direct consequence of abdominal septic complications to intestinal surgery.

Although novel therapies such as small bowel transplantation, intestinal lengthening surgery and intestinal growth factor therapy are appropriate in a minority of patients, the majority of people with long-term intestinal failure depend on lifelong parenteral nutrition for their survival. In order to minimise the risk for this life-altering complication, a better understanding of the mechanisms of IF in CD is required.

We therefore performed a detailed study of all consecutive patients admitted to our national intestinal failure unit with an underlying diagnosis of CD from the year 2000. Furthermore, we studied long-term outcomes, including nutritional autonomy and mortality, to characterise the prognosis of IF in CD.

## 2. Materials and Methods

### 2.1. Study cohort

Consecutive patients aged 16 years or older with CD, admitted to a national intestinal failure unit in England between January 2000 and January 2018, were identified from a prospectively collected institutional database and included in this study. The current European Society for Clinical Nutrition and Metabolism [ESPEN] definition of IF was used.^2^ Patients with parenteral nutrition lasting less than 12 months were excluded.

Details of intestinal failure management and outcomes until January 31, 2019, were collected. The majority of patients received a single-lumen tunnelled central venous catheter. Parenteral nutrition was administered either by the patients, their relatives, or trained nursing staff using a standardised protocol in accordance with ESPEN guidance.^13^ Most patients received overnight infusions during the minimum number of nights required to meet their nutritional needs. Patients were encouraged to have oral nutrition when feasible. Parenteral lipids were delivered once or twice per week and lipid dosing was generally limited to 1 g/kg/day. In patients with diabetes, lipids were the main source of calories where required.

Patients were reviewed at least 6-monthly in clinic or by videolink. Patients in whom parenteral nutrition was discontinued were monitored for at least another 6 months. A small number of patients had two periods of IF lasting 12 months or more. In those patients, data for the first episode were recorded only, so that each patient was only entered once in the study.

### 2.2. Data collection

The cohort was studied longitudinally. Data on demographics, anatomy, parenteral nutrition, and outcomes were collected from the prospective institutional intestinal failure database. The cause of IF was independently assessed in each case by two of three authors [EN, HK, and MS]. In each case, the cause which led directly to IF was identified. Disagreements were resolved by joint case review.

The following four direct causes were defined: disease activity, postoperative abdominal sepsis, uncomplicated bowel resection, and proximal stoma. Disease activity was considered the cause in patients who developed IF as a direct consequence to severe inflammatory, stricturing, and/or penetrating Crohn’s disease. The other three main causes were all postoperative. Postoperative abdominal sepsis was considered the cause when postoperative abdominal or pelvic sepsis, or procedures required to treat such sepsis, resulted in IF. Three subcategories to abdominal sepsis were defined: postoperative sepsis related to a new bowel anastomosis; postoperative fistulation between bowel and skin or wound unrelated to an anastomosis; and postoperative abscess unrelated to an anastomosis.

Uncomplicated bowel resection was considered the cause of IF when IF developed after a bowel resection that, although uncomplicated, resulted in loss of absorptive function so that parenteral administration of fluids or nutrition was required. Proximal stoma was considered the cause when IF occurred after a stoma was fashioned proximal to a significant amount of bowel, for example to protect multiple distal anastomoses and/or strictureplasties or when undiverted anastomosis was deemed unsafe due to risk factors for poor healing at the time of surgery.

The date of nutritional autonomy was defined as the date when regular parenteral nutrition was permanently stopped. Patients in whom parenteral nutrition was subsequently restarted were not considered nutritionally autonomous.

Reconstructive surgery was defined as elective abdominal surgery at the Intestinal Failure Unit aiming to improve gut function. Intestinal failure-associated liver disease was defined as an increase to 1.5 times the upper limit of normal or more for >6 months of blood concentration levels of at least two of the three following measurements: alkaline phosphatase, gamma‐glutamyl transferase, and bilirubin.^14^ The Unit was alerted to all deaths occurring during the period of follow-up at Salford Royal Hospital. Deaths occurring subsequent to discharge from the hospital were not recorded, and hence these data were censored at date of discharge from follow-up.

### 2.3. Statistical methods

Nominal and ordinal data are presented as proportions, and continuous data as means ± standard deviation [SD] or medians [range] as appropriate. Differences between groups in nominal or ordinal data were assessed by the chi square test. Differences between groups in continuous data were compared using one-way analysis of variance [ANOVA] with Tukey’s honestly significant difference [HSD] post-hoc tests when appropriate.

Time from diagnosis of CD and onset of IF was found to be log-normally distributed. These data were therefore log-transformed prior to ANOVA. Statistical analysis was performed using the JMP software package [JMP 14.0 for Mac OS X, SAS, Cary, NC, USA].

Kaplan-Meier curves were generated for the incidence of nutritional autonomy and death. The censoring date was January 31, 2019. Simple and multivariable Cox regression analyses were performed to identify factors associated with nutritional autonomy and death. Where data were missing for variables, analyses were undertaken with data available and the number of missing data points was indicated.

Trends over time were analysed by comparing two time periods chosen to include an equal number of patients commenced on HPN over the 18-year period.

The STROBE guidelines for reporting of cohort studies were followed [www.strobe-statement.org].

## 3. Results

### 3.1. Patient characteristics

In all 121 patients [67 women and 54 men] were admitted between January 2000 and January 2018 with IF lasting more than 12 months and a diagnosis of CD. The median [range] age at diagnosis of CD was 26 [7–85] years. Intestinal failure developed 14 [0–50] years later at an age of 48 [16–86] years. The majority had an ileal stricturing phenotype at diagnosis of CD [Table 1].

**Table 1.** Phenotypic characteristics at diagnosis of Crohn’s disease in 121 people who later developed intestinal failure.

	Number [%]
Age at diagnosis	
A1 [≤16 years]	22 [19%]
A2 [17–40 years]	63 [53%]
A3 [>40 years]	33 [28%]
Location	
L1 [ileal]	54 [46%]
L2 [colonic]	25 [21%]
L3 [ileo-colonic]	37 [31%]
L4 [upper gastrointestinal]	2 [1%]
Behaviour	
B1 [inflammatory]	33 [28%]
B2 [stricturing]	63 [53%]
B3 [penetrating]	22 [19%]

Preceding the onset of IF, 101 patients [84%] had received corticosteroid therapy, 82 [69%] immunomodulator therapy, and 57 [48%] biologic therapy; 118 [98%] patients had undergone abdominal surgery for Crohn’s disease at some point prior to onset of IF, and three patients never had surgery for CD before IF. On admission, 26 [21%] were current smokers, 31 [26%] previous smokers, and 64 patients [53%] had never smoked.

### 3.2. Causes of intestinal failure

#### 3.2.1.Main mechanisms

In over half of patients with IF and Crohn’s disease, IF was caused by an abdominal septic complication to abdominal surgery [Table 2]. Uncomplicated bowel resection was the cause in less than one-third of IF cases. In a small number, proximal diversion caused IF and another small group developed IF secondary to primary disease activity rather than a recent operation.

**Table 2.** Direct causes of intestinal failure [IF] in 121 people with Crohn’s disease [CD].

	Primary disease	Postoperative abdominal sepsis	Uncomplicated bowel resection	Proximal diversion	*p*
Number [%]	14 [12]	62 [51]	38 [31]	7 [6]	
Gender [male:female]	8:6	29:33	13:25	4:3	0.37
Age at onset of IF [years]	48 [18–63]	43 [16–76]	49 [26–78]	52 [19–86]	0.33
Time from CD to IF [years]	13 [1–39]	10 [0–50]^a^	21 [1–47]	15 [1–41]	0.045
Operations prior to IF	2 [0–6]^b,c^	3 [1–8]	4 [1–7]	4 [1–5]	0.003
Small bowel length at IF [cm]	315 [150–400]^d,e,f^	135 [30–400]	120 [12–270]	120 [60–180]	<0.0001
Colon in continuity at IF [%]	7 [50]	18 [29]	19 [50]	0 [0]	0.022

Medians [range]. All post-hoc Tukey’s HSD test.

^a^ *p* = 0.0034 vs uncomplicated bowel resection.

^b^ *p* = 0.0273 vs uncomplicated bowel resection.

^c^ *p* = 0.0152 vs postoperative abdominal sepsis.

^d^ *p* <0.0001 vs postoperative abdominal sepsis.

^e^ *p* <0.0001 vs uncomplicated bowel resection.

^f^ *p* <0.0001 vs proximal diversion.

The interval between diagnosis of CD and IF onset differed between the aetiological groups [Table 2]. Patients who developed IF due to uncomplicated bowel resection did so after more than twice as long as patients who had a postoperative septic complication. Patients who developed IF due to primary disease activity had undergone the lowest number of abdominal operations before IF. Among all 121 patients, the median length of small bowel in continuity at onset of IF was 150 [12–400] cm, with patients who developed IF due to primary disease having the longest length of small bowel in continuity, although the range was wide in all four groups [Table 2].

Overall, 44 [36%] of patients had their colon in continuity at IF onset. By definition, none of the patients with a proximal diversion had their colon in continuity, and only a minority of patients with postoperative abdominal sepsis did so [Table 2]. None of the components of the Montreal classification at diagnosis of CD differed between groups [data not shown].

#### 3.2.2. Groups with intestinal failure after surgery

Thus, in 107 patients [88%] IF followed immediately after an abdominal operation. The operation that led to IF was elective in 66/106 [62%] and emergency in 40/106 [38%] [missing data, *n* = 1].

Among the 62 patients with postoperative abdominal sepsis as the index event, sepsis occurred in relation to an anastomosis in 32, in 25 an enteric fistula, and in 5 an intra-abdominal abscess developed unrelated to an anastomosis.

Of the 32 patients with postoperative sepsis related to an anastomosis, the anastomosis had been formed during an emergency procedure in 11 [34%]. Furthermore, at the time of surgery, ongoing corticosteroid therapy was documented in 12 of the 32 patients, anti-tumour necrosis factor [TNF] therapy in two patients, and immunomodulator therapy in three patients. Three patients had an intra-abdominal abscess documented at the time of formation of the anastomosis, and three had documented significant weight loss prior to surgery. Fifteen [47%] of the 32 patients with anastomosis-related sepsis had documentation of at least one of the risk factors of ongoing steroid therapy, anti-TNF therapy, intra-abdominal abscess, or significant preoperative weight loss.

### 3.3. Outcomes of intestinal failure

**Figure 1.**
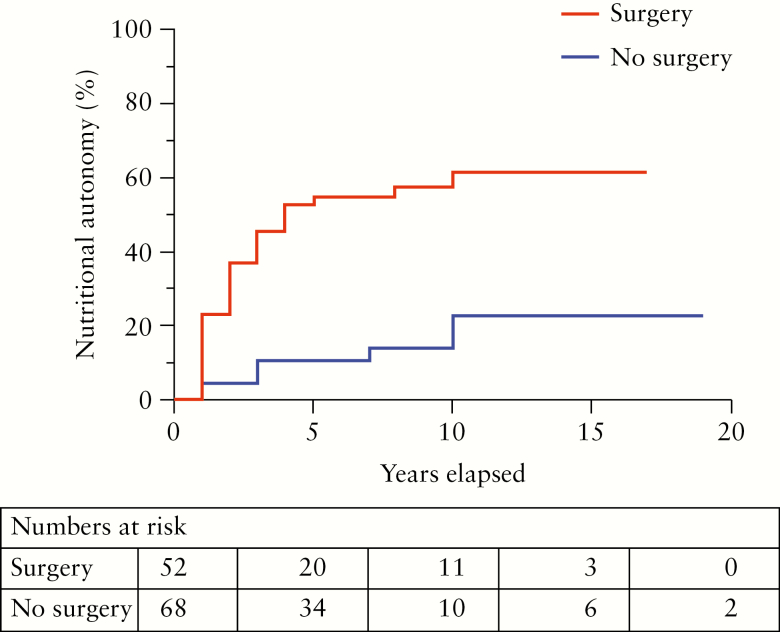
Cumulative incidence of nutritional autonomy in 121 patients with intestinal failure and Crohn’s disease who did [red, *n* = 52] and did not [blue, *n* = 68][missing data, *n* = 1] undergo reconstructive surgery; *p <0.0001.

The median duration of parenteral nutrition at data collection was 4.5 [1–20] years. At the time of data collection, 40 of the 121 [33%] had regained nutritional autonomy [Figure 1]. A total of 52 patients underwent reconstructive surgery, 31 [60%] of whom had regained nutritional autonomy at data collection.

Of 69 patients who did not undergo reconstructive surgery, nine [13%] regained nutritional autonomy at data collection. On univariable analysis, no predictor of nutritional autonomy could be identified, such as cause of IF, small bowel length, or colon in continuity [data not shown].

On Kaplan-Meier analysis, 61% of operated patients and 22% of patients not operated upon regained nutritional autonomy 10 years after onset of IF, with no further patients after this time [Figure 1]. Overall, 40% of all 121 patients had regained nutritional autonomy after 10 years, on Kaplan-Meier analysis.

**Figure 2.**
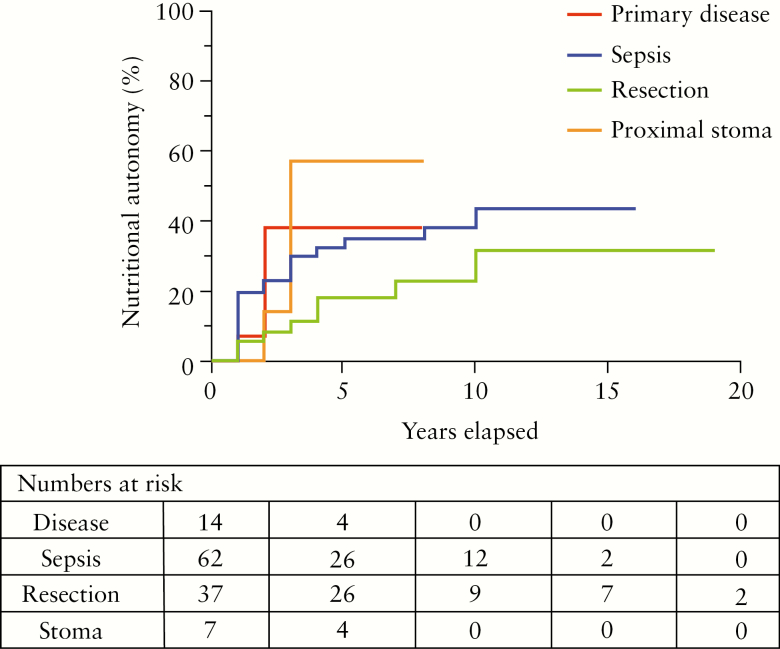
Cumulative incidence of nutritional autonomy in 121 patients with intestinal failure and Crohn’s disease due to primary disease [*n *= 14], postoperative abdominal sepsis [*n* = 62], uncomplicated bowel resection [*n* = 37], and proximal diversion [*n* = 7][missing data, *n* = 1]. Difference between groups, *p =* 0.26.

The cause of IF did not influence the likelihood of regaining nutritional autonomy over time, on Kaplan-Meier analysis [Figure 2, *p* = 0.26]. More patients with proximal diversion appeared to regain autonomy, and more rapidly, than did patients with other aetiologies, but numbers were low [Figure 2].

Intestinal failure-associated liver disease developed in 17 [14%] patients. At the time of data collection, 81/121 [67%] had quiescent Crohn’s disease, 39/121 [32%] had disease requiring medical therapy, and one [1%] was awaiting surgery for complications to Crohn’s disease.

**Figure 3.**
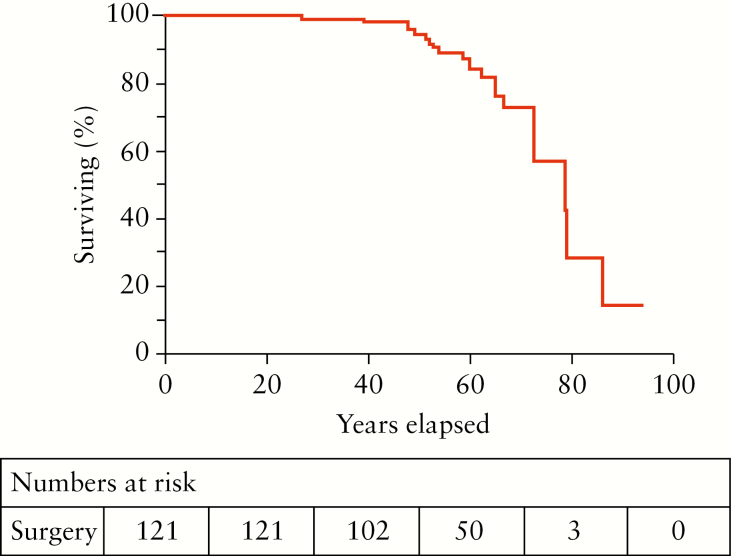
Cumulative survival in 121 patients with intestinal failure and Crohn’s disease.

At the time of data collection, 21/121 [17%] patients were reported deceased. Kaplan-Meier analysis showed a projected mean [SD] age of death of 74 [2] years [Figure 3].

### 3.4. Trends over time

The median year of admission was 2011. Hence, an early period was defined as date of presentation between January 2000 and December 2010 [*n* = 60], and a late period as dated from January 2011 until January 2018 [*n* = 61]. Treatment with steroids before onset of IF decreased between the early and late time periods [92% vs 77%, respectively, *p* = 0.024] and, conversely, the use of biologic agents increased [38% vs 57%, *p* = 0.044].

**Figure 4.**
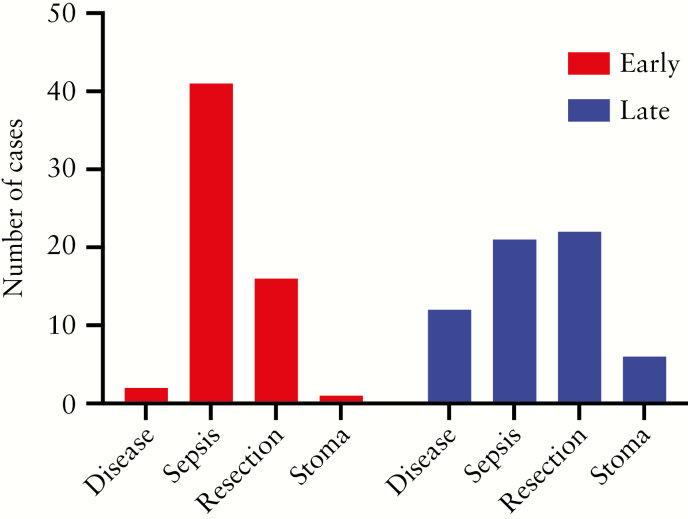
Direct causes of intestinal failure in 121 patients with Crohn’s disease during 2000–2010 [*n* = 60] vs 2011–2018 [*n* = 61]. Disease, primary disease; sepsis, postoperative abdominal sepsis; resection, uncomplicated resection; stoma, proximal stoma. Chi square test *p* <0.001.

Intestinal failure occurred earlier in the disease process in the early vs the late period: 10 [0–50] vs 16.5 [1–47] years [*p* = 0.030]. The direct cause of IF changed across the two time periods [*p* <0.001, Figure 4]. In particular, the proportion of patients who had IF due to postoperative abdominal sepsis decreased from the early to the late period [68% vs 34%], whereas the proportion of patients with primary disease activity and proximal stoma as the cause of IF increased [3% vs 20% and 2% vs 10%, respectively].

**Figure 5.**
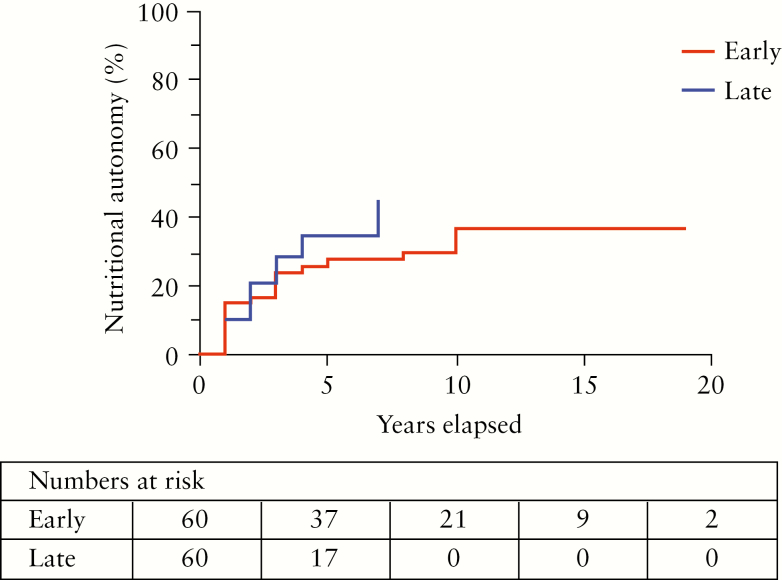
Cumulative incidence of nutritional autonomy in 121 patients with intestinal failure and Crohn’s disease comparing patients admitted 2000–2010 [early, *n *= 60] vs 2011–2018 [late, *n* = 60][missing data, *n* = 1]; *p* = 0.59.

The cumulative incidence of nutritional autonomy as assessed by Kaplan-Meier analysis did not differ between the time periods [*p =* 0.59, Figure 5].

## 4. Discussion

In this large, longitudinal cohort study, the mechanisms and long-term outcomes of intestinal failure in Crohn’s disease were studied for the first time. Intestinal failure can develop in Crohn’s disease through a number of mechanisms: cumulative loss of small bowel through multiple resections, enterocutaneous fistulation, and malabsorption are often mentioned, with multiple resections being the mechanism that is most prominently quoted in textbooks and current clinical guidelines.^7–9^

However, with the exception of our previous smaller study,^12^ published studies of IF in CD have exclusively presented associations between baseline characteristics such as phenotype and the risk of developing IF.^15–17^ The mechanisms that led directly to IF have not been studied. An understanding of those mechanisms is necessary to develop strategies to lessen the risk of this life-altering complication.

In the present study, where the mechanism of IF was determined in 121 patients by detailed assessment of events in the referring centre, we demonstrated that in 51% of cases, the direct cause was abdominal sepsis complicating an abdominal operation. Intestinal resection was the direct cause in 31%. Primary disease activity and proximal diversion were less common direct causes. These findings confirm and expand upon those of our previous smaller study, where 61% of cases were due to postoperative abdominal sepsis and 22% due to intestinal resections.^12^

In 32 of the 62 patients with postoperative abdominal sepsis, the source of sepsis was related to an intestinal anastomosis. In nearly half of those patients, at least one of the risk factors associated with an increased risk for anastomotic dehiscence was documented in available records. Those complications were concurrent steroid therapy, recent anti-TNF therapy, intra-abdominal abscess, or significant preoperative weight loss. This finding may be an underestimate, due to the retrospective nature of data retrieval.

Strategies to reduce postoperative abdominal sepsis may therefore be important to prevent IF in CD. Contemporary large observational studies suggest that the rate of abdominal septic complications in Crohn’s disease in current surgical practice is around 9–19%,^18–20^ emphasising the need for such strategies. Postoperative abdominal sepsis often results from fashioning bowel anastomoses in the presence of risk factors for poor healing.^21–26^ The risk of anastomotic dehiscence in Crohn’s disease can be reduced either by structured preoperative optimisation targeting such risk factors,^27–31^ or avoiding an anastomosis if optimisation is not feasible.

The recognised risk factors that are targeted in preoperative optimisation include systemic steroid treatment, ^19,21–23,32^ recent weight loss,^23,32^ intraabdominal abscess,^24–26^ and smoking.^32–34^ Whether recent administration of anti-tumour necrosis factor medication is associated with postoperative morbidity in Crohn’s disease is currently under investigation. The largest prospective study that has been published found that anti-TNF exposure, after adjustment for disease severity and other confounders, increased overall and intra-abdominal septic morbidity.^25^ The largest published meta-analysis similarly found increased overall and infectious morbidity in patients exposed to anti-TNF therapy, after adjustment for disease severity and other confounders.^35^ Based on best available evidence, therefore, recent administration of anti-TNF agents was included as a risk factor in the present analysis. Additional studies are under way and will further define this risk.

When optimisation is not possible, we instead exteriorise the bowel as double-barrelled stoma, which entirely removes the risk of anastomotic dehiscence.^36^ Proximal diversion of a primary anastomosis has also been described, reducing this risk by 55% in a large series.^32^

Thirty patients developed IF due to postoperative abdominal sepsis unrelated to an anastomosis, in most cases manifesting as an enterocutaneous or an entero-atmospheric fistula. Mechanisms were not further subclassified but included iatrogenic bowel injury, fistulation due to an open abdomen, and intraoperative contamination. Optimal surgical technique may reduce such complications. For example, adhesiolysis in dense adhesions is known to be associated with inadvertent enterotomy, itself associated with increased morbidity and mortality,^37,38^ and we favour knife dissection in this situation.

On Kaplan-Meier analysis, 40% of patients regained nutritional autonomy during long-term follow-up. As expected, patients who underwent surgery aiming to restore gastrointestinal continuity achieved nutritional autonomy to a much higher degree than patients who did not [60% vs 13%]. All patients who achieved nutritional autonomy did so by 10 years after onset of IF. Patients who weaned parenteral nutrition without surgery likely did so by a combination of intestinal and metabolic adaptation and dietary optimisation.^39^ We were not able to identify factors associated with non-operative weaning, likely due to the low number of patients who did wean. In our 30-year study of intestinal failure from all causes, 30% of people on long-term home parenteral nutrition achieved nutritional autonomy within 15 years, in line with the present findings in CD.^3^ In the future, recently developed therapies including growth factor therapy,^40,41^ intestinal lengthening surgery,^42^ and intestinal transplantation^43–45^may further improve the prognosis in IF associated with CD.

Some changes over the 18-year study period were observed. In particular, the proportion of postoperative abdominal sepsis as a cause of IF appeared to decrease, whereas the use of proximal stomas appeared to increase. Increased specialisation of care for inflammatory bowel disease and greater awareness of perioperative risks in Crohn’s disease, with more judicious use of anastomosis, may be contributing factors.

The present study has unique strengths, including the largest published cohort of its kind and prospective and nearly complete follow-up, but some weaknesses are inherent in retrospective studies. Assessment of the most direct cause of IF was subjective, although bias was minimised by two investigators independently assessing each case. Another possible source of error was a lack of detail available on events occurring in each patient’s local hospital. This includes deaths occurring subsequent to discharge from follow-up at the Intestinal Failure Unit. In the wider context, this study cannot comment on the incidence of IF in CD, as the denominator [the prevalence of CD in the referring regions] is unknown. Similarly, trends in the incidence of IF could not be analysed. Larger longitudinal cohort studies are needed to address these important issues.^5,6^

In conclusion, IF is a severe complication of CD, with 60% of patients permanently dependent on parenteral nutrition. The most frequent event leading directly to IF was a septic complication following abdominal surgery, rather than loss of small bowel due to multiple resections. In many cases, such sepsis followed intestinal anastomosis in the presence of significant risk factors for anastomotic dehiscence. A reduced need for abdominal surgery, an increased awareness of perioperative risk factors, and structured pre-operative optimisation may reduce the incidence of IF in CD.

## Funding

This work received no external financial support. The data were generated as part of the routine work of Irving National Intestinal Failure Unit, Salford Royal Hospital.

## Conflict of Interest

The authors declare that there are no conflicts of interest.

## Author Contributions

MS: concept and design, acquisition of data, analysis and interpretation, drafting or revising, final approval. HK: acquisition of data, analysis and interpretation, drafting or revising, final approval. EM: acquisition of data, analysis and interpretation, drafting or revising, final approval. AT: concept and design, acquisition of data, drafting or revising, final approval. AA: concept and design, acquisition of data, drafting or revising, final approval. GC: concept and design, analysis and interpretation, drafting or revising, final approval. SL: concept and design, analysis and interpretation, drafting or revising, final approval.
